# Navigating Human Epidermal Growth Factor Receptor 2 (HER2) Conversion: Insights From Recurrent Breast Cancer

**DOI:** 10.7759/cureus.61305

**Published:** 2024-05-29

**Authors:** Mohammad Z Al-Bdour, Rula Al-Shimi, Mohammad J Al-Rifai, Hani El-Taani, Abdulqadir J Nashwan

**Affiliations:** 1 Oncology, Faculty of Medicine, Jordan University of Science and Technology, Amman, JOR; 2 Internal Medicine, Jordan University of Science and Technology, Amman, JOR; 3 Oncology, King Abdullah University Hospital, Irbid, JOR; 4 Nursing & Midwifery Research, Hamad Medical Corporation, Doha, QAT

**Keywords:** oncology, biomarker assessment, treatment adjustments, her2 receptor conversion, recurrent breast cancer

## Abstract

Recurrent breast cancer presents clinical challenges due to its dynamic nature. Turning human epidermal growth factor receptor 2 (HER2) status from negative to positive upon recurrence is a rare but clinically significant phenomenon that can impact treatment decisions. We present the case of a 63-year-old female initially diagnosed with stage IIIA breast cancer, characterized as HER2-negative. However, upon recurrence eight years later, the patient exhibited HER2 conversion, indicating a positive status. Subsequent treatment adjustments were made based on this new HER2-positive status, leading to complete remission. HER2 conversion underscores the dynamic nature of tumor biology in recurrent breast cancer. This case highlights the importance of re-biopsy for accurate biomarker assessment and the necessity of personalized treatment strategies based on current molecular profiles. Understanding and recognizing HER2 conversion in recurrent breast cancer is crucial for optimizing patient outcomes and guiding clinical management decisions. Further research is warranted to elucidate the frequency and clinical implications of HER2 conversion in recurrent breast cancer.

## Introduction

Breast cancer is the most prevalent cancer among women globally [1], showing significant health and therapeutic challenges across diverse populations. Regardless of advancements in early detection and treatment modalities, a significant proportion of patients experience disease recurrence, either locoregional or distant metastasis, contributing to ongoing morbidity and mortality associated with the disease [2]. This recurrence not only marks an important point in a patient’s clinical journey but also brings to light the dynamic nature of breast cancer biology, especially in the context of tumor heterogeneity and biomarker evolution over time. The management of recurrent metastatic breast cancer (RMBC) requires a nuanced understanding of the tumor’s biological characteristics, which may develop significantly from the initial diagnosis [3]. Biomarkers such as hormone receptors (HRs) for estrogen (ER) and progesterone (PgR), along with the human epidermal growth factor receptor 2 (HER2), are very important in guiding treatment decisions. They influence the choice of hormonal therapies, targeted agents, and chemotherapy regimens, directly impacting patient outcomes [4]. The traditional dependence on initial biopsy results for treatment planning in recurrent cases is increasingly challenged by evidence of biomarker alteration in disease recurrence, an observation attributed to tumor clonal development, selective therapeutic pressures, and the inherent heterogeneity of breast cancer [5]. The important recommendation for repeat biopsy in cases of RMBC, as accepted by clinical guidelines, is predicated on the potential for changes in these critical biomarkers [6]. Such moves can radically alter the therapeutic landscape, transitioning patients into more effective, targeted treatment options that were previously not indicated. However, despite these recommendations, the real-world clinical impact of re-biopsy and biomarker reassessment remains a topic of active discussion. Suspicion persists regarding the prevalence of biomarker conversion and whether these changes, when identified, translate into meaningful clinical benefits for patients.

This case report focuses on the clinical journey of a patient with RMBC whose treatment trajectory was profoundly influenced by the reassessment of HER2 status upon recurrence. Through this point, we explore the broader implications of re-biopsy in recurrent breast cancer and aim to contribute to the ongoing discourse on its value and impact on clinical outcomes.

## Case presentation

In September 2012, a 63-year-old female was diagnosed with stage IIIA right breast cancer which was characterized as grade 2 invasive ductal carcinoma. Immunohistochemical (IHC) examination at diagnosis showed the tumor to be positive for ER, moderately positive for PgR, and weakly positive for HER2 (IHC 1+), with no HER2 amplification detected via fluorescence in situ hybridization (FISH). After a modified radical mastectomy and sentinel lymph node biopsy, the patient was treated with adjuvant chemotherapy consisting of four cycles of Taxol (paclitaxel 175 mg/m^2^) and 25 cycles of radiotherapy After treatment, she entered a period of monitoring without additional therapy.

Eight years later, the patient began suffering from left-sided breast pain and noticed a neck mass. Physical examination revealed enlargement of three cervical lymph nodes. Positron emission tomography-computed tomography (PET-CT) scans confirmed the presence of metastatic disease in the posterior and deep cervical lymph nodes.

Ultrasonography-guided fine-needle aspiration (FNA) cytology of these nodes confirmed metastasis from breast cancer with the ER, PgR, and HER2 status unchanged from the initial diagnosis. The mammogram and ultrasound of the left breast were considered Breast Imaging Reporting and Data System 2 (BIRADS 2), which indicates benign findings. No additional metastases were identified beyond the lymph nodes.

The patient was treated with tamoxifen and palbociclib (125 mg), achieving complete remission as evidenced by the resolution of lymph node swelling on follow-up PET-CT scans.

**Figure 1:**
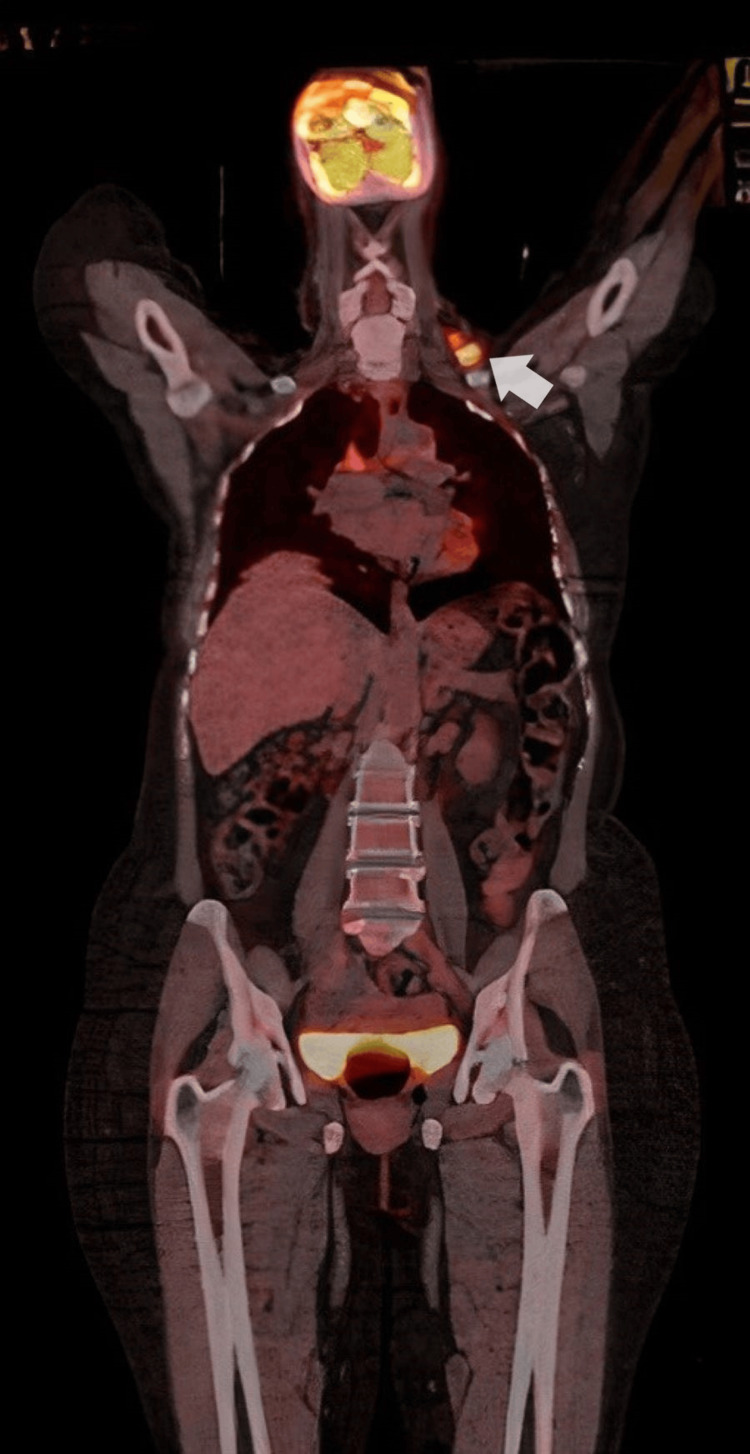
Positron emission tomography-computed tomography scan reveals a hyperintense lymph node in the supraclavicular region.

**Figure 2:**
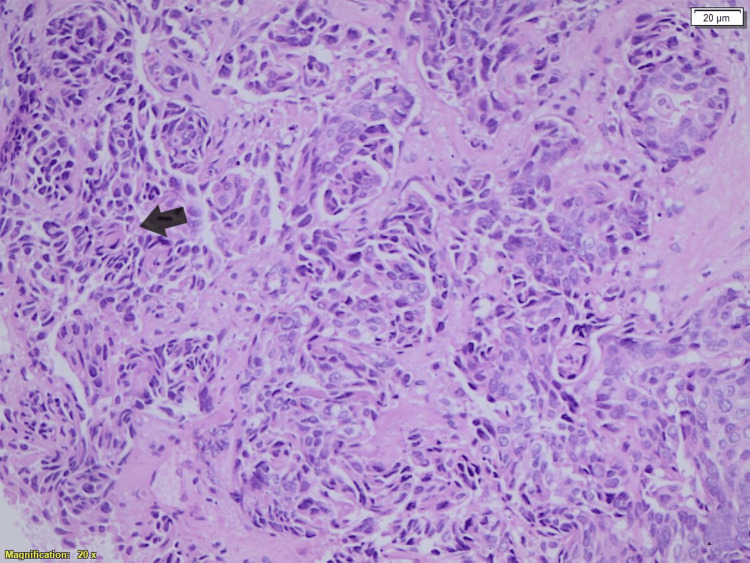
Hematoxylin and eosin staining of breast tissue shows malignant cells are infiltrated and arranged in a single-file, cord-like growth pattern.

**Figure 3:**
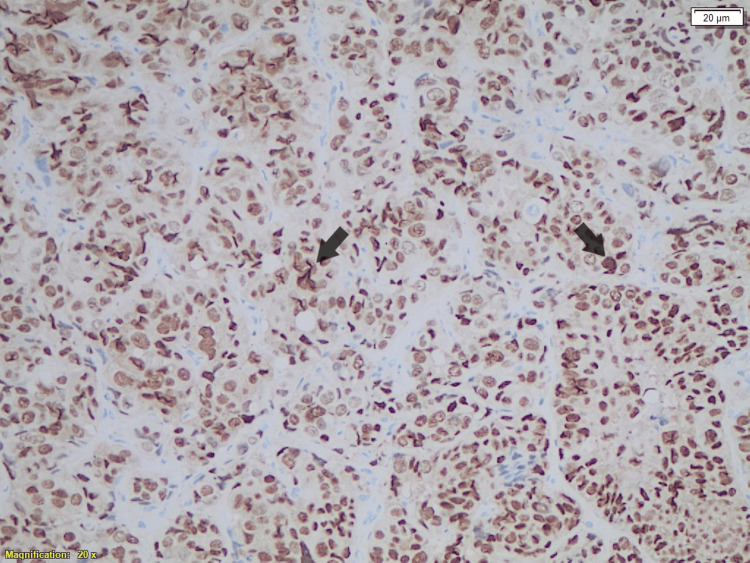
Immunohistochemistry analysis of the breast metastasis showing progesterone receptor-positive cells from the breast tumor.

**Figure 4:**
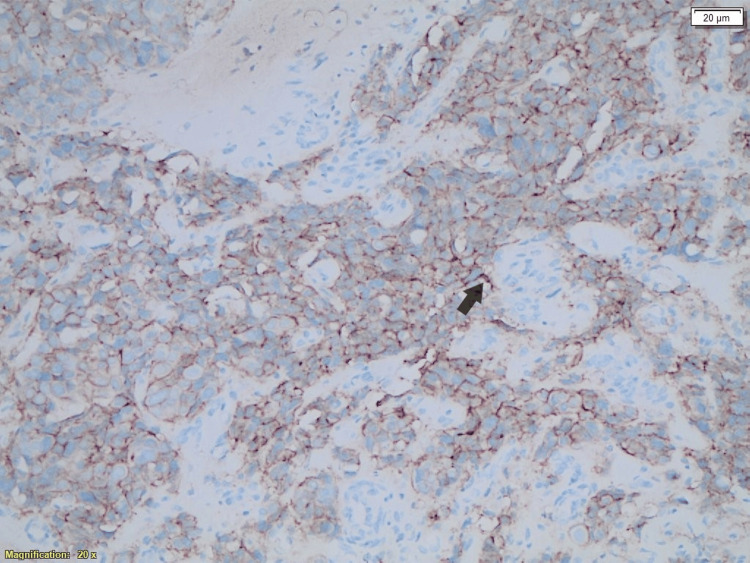
Immunohistochemistry analysis of the breast metastasis showing HER2/neu-positive cells from the breast tumor.

**Figure 5:**
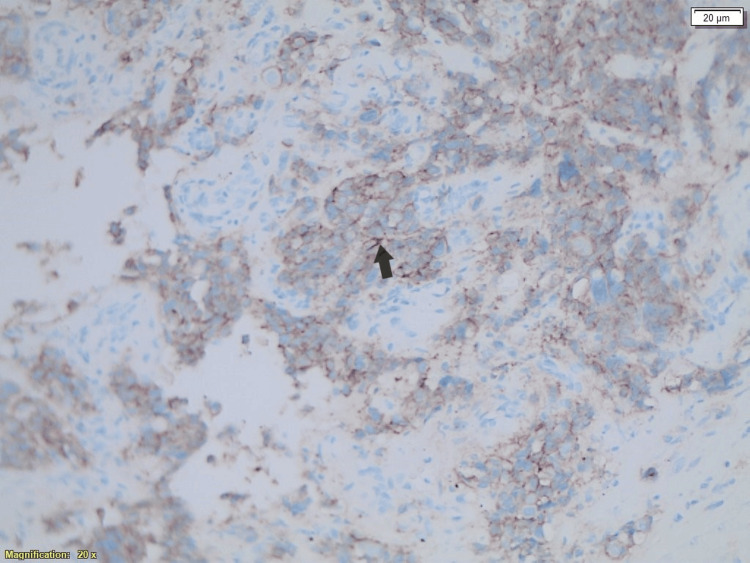
Immunohistochemistry analysis of the breast metastasis showing CK20-positive cells from the breast tumor.

In December 2022, the patient presented with complaints of a neck lump. A PET-CT scan demonstrated swollen supraclavicular lymph nodes (Figure 1). A biopsy and subsequent histological analysis confirmed breast cancer recurrence (Figure 2). Cytological analysis from FNA samples revealed breast cancer metastasis, with a notable change: the HER2 status had converted to positive (IHC 2+), as confirmed by HER2 amplification on FISH analysis, with a positive PgR and cytokeratin 20 (CK20) (Figures 3-5). This biomarker conversion led to a significant shift in the treatment approach. The patient commenced a regimen of pertuzumab (420 mg), trastuzumab (6 mg/kg), and docetaxel (75 mg/m^2^).

The treatment was tolerated, with the most significant adverse events being alopecia (grade 1) and neutropenia (grade 2). Following cycles of this treatment, a follow-up PET-CT scan showed complete remission of the supraclavicular lymph node metastases. The patient was assessed to have achieved a complete clinical response according to Response Evaluation Criteria in Solid Tumors (RECIST) criteria and has since been in complete remission.

## Discussion

The development of biomarkers in recurrent breast cancer, especially the conversion of HER2 status from negative to positive, as observed in our case, underscores the dynamic nature of tumor biology over the disease course [7]. Although relatively rare, this situation has profound implications for the treatment and prognosis of patients with RMBC [8]. The case of our 63-year-old patient explains an important example where re-biopsy led to a significant alteration in the treatment strategy, ultimately contributing to a remarkable clinical outcome.

Significance of HER2 status conversion

HER2-positive tumors are known to be more aggressive but are docile to targeted therapies that have significantly improved outcomes for patients with this breast cancer subtype [9]. As observed in this case, the conversion of HER2 status from negative to positive in the context of metastasis presents an opportunity to utilize HER2-targeted therapies that were not previously indicated. This aligns with findings from several studies indicating that the reassessment of biomarkers at the time of recurrence can reveal new actionable targets, thereby enabling the personalization of therapy based on the current tumor biology rather than relying solely on the initial diagnostic profile [10].

Implications for clinical practice

The clinical impact of re-biopsy in RMBC extends beyond identifying biomarker status change; it directly influences treatment decisions, potentially leading to more effective and tailored therapeutic approaches [11]. This case supports the importance of guideline recommendations advocating for repeat biopsies in the setting of breast cancer recurrence or metastasis. In addition, it highlights the necessity for clinicians to remain careful about the possibility of biomarker conversions and to embrace the evolving landscape of precision oncology, where treatment decisions are increasingly driven by real-time tumor biology.

Despite the potential benefits, implementing routine re-biopsy in clinical practice is challenging. These include procedural risks, patient discomfort, and the feasibility of obtaining sufficient tissue samples from metastatic sites. Additionally, the cost-effectiveness and impact on overall survival and quality of life require further research. It is crucial for future studies to address these considerations, providing clearer guidance on the optimization of re-biopsy strategies in the management of RMBC.

Our case contributes to the growing body of evidence supporting the clinical value of re-biopsy in RMBC, especially in HER2 status conversion. It emphasizes the need for ongoing research to better understand the mechanisms underlying biomarker development and explore the full spectrum of therapeutic implications. Further studies are warranted to detect the frequency of such conversions to identify biomarker change predictors and quantify the impact of tailored treatments on patient outcomes.

## Conclusions

Our case highlights the important role of re-biopsy in the management of recurrent metastatic breast cancer, especially underscoring the potential for biomarker conversion throughout the disease. The observed conversion of HER2 status from negative to positive in a patient with RMBC not only challenges the traditional reliance on initial diagnostic profiles for treatment planning but also supports the dynamic nature of breast cancer biology. This case focuses on the importance of re-evaluating tumor characteristics at the time of recurrence, facilitating the adoption of personalized treatment strategies tailored to the tumor’s current molecular landscape. The implications of our findings are twofold. Clinically, they support the integration of a repeat biopsy into standard practice for patients with recurrent disease to ensure that therapeutic decisions are informed by the most accurate and up-to-date information. From a research perspective, this case contributes to the growing evidence base advocating for a more nuanced understanding of biomarker evolution in breast cancer, which could pave the way for advancements in the precision oncology approach. Moreover, this case compellingly illustrates the clinical impact and utility of re-biopsy in the era of personalized medicine, advocating for its broader implementation in the management of recurrent metastatic breast cancer. It calls for continued vigilance and adaptability in treatment planning, ensuring patients benefit from the latest insights and innovations in cancer care.
